# Characterization of Antifungal Lipopeptide Biosurfactants Produced by Marine Bacterium *Bacillus* sp. CS30

**DOI:** 10.3390/md17040199

**Published:** 2019-03-29

**Authors:** Shimei Wu, Ge Liu, Shengnan Zhou, Zhenxia Sha, Chaomin Sun

**Affiliations:** 1 College of Life Sciences, Qingdao University, Qingdao 266071, China; shimeiwu2016@126.com (S.W.); 18363995124@163.com (S.Z.); 2 CAS Key Laboratory of Experimental Marine Biology, Institute of Oceanology, Chinese Academy of Sciences, Qingdao 266071, China; liug878@163.com; 3 Laboratory for Marine Biology and Biotechnology, Qingdao National Laboratory for Marine Science and Technology, Qingdao 266071, China; 4 Center for Ocean Mega-Science, Chinese Academy of Sciences, Qingdao 266071, China

**Keywords:** lipopeptide, surfactin, *Bacillus*, antifungal, reactive oxygen species

## Abstract

This study was initiated to screen for marine bacterial agents to biocontrol *Magnaporthe grisea*, a serious fungal pathogen of cereal crops. A bacterial strain, isolated from the cold seep in deep sea, exhibited strong growth inhibition against *M. grisea*, and the strain was identified and designated as *Bacillus* sp. CS30. The corresponding antifungal agents were purified by acidic precipitation, sequential methanol extraction, Sephadex LH-20 chromatography, and reversed phase high-performance liquid chromatography (RP-HPLC), and two antifungal peaks were obtained at the final purification step. After analysis by mass spectrometry (MS) and tandem MS, two purified antifungal agents were deduced to belong to the surfactin family, and designated as surfactin CS30-1 and surfactin CS30-2. Further investigation showed that although the antifungal activity of surfactin CS30-1 is higher than that of surfactin CS30-2, both of them induced the increased generation of reactive oxygen species (ROS) and caused serious damage to the cell wall and cytoplasm, thus leading to the cell death of *M. grisea*. Our results also show the differences of the antifungal activity and antifungal mechanism of the different surfactin homologs surfactin CS30-1 and surfactin CS30-2, and highlight them as potential promising agents to biocontrol plant diseases caused by *M. grisea*.

## 1. Introduction

With the emerging resistance of pathogens to commonly used antibiotics or fungicides, the discovery and development of new alternative chemicals is of the utmost importance. Lipopeptides are natural small cyclic or linear compounds synthesized by various microorganisms, which consist of hydrophobic long alkyl chains and hydrophilic polypeptides [1]. Due to their amphipathic characteristics, lipopeptides can induce the formation of pore and ion channels in lipid bilayer membranes and cause less pathogen resistance compared with traditional antibiotics or fungicides. In addition, lipopeptides are more environment-friendly because of their low toxicity and high biodegradability, and also possess high stability towards extreme temperature, pH, and salinity, thus becoming an alternative to ordinary chemical agents [2,3,4]. Furthermore, lipopeptides exhibit a variety of activities in anti-bacterial, anti-fungal, anti-viral, anti-inflammatory, and anti-cancer aspects, indicating that lipopeptides have high application potentialities in chemical, agricultural, pharmaceutical, and food industries [5,6,7].

*Bacillus* sp., such as *B. subtilis*, *B. cereus*, *B. licheniformis*, and *B. amyloliquefaciens*, have been reported as the major producers of lipopeptides [1,8,9,10]. Lipopeptides produced by *Bacillus* are synthesized by large multi-enzyme, non-ribosomal peptide synthetases (NRPSs), which lead to a remarkable heterogeneity among the lipopeptides with regards to the type and sequence of amino acid residues, the nature of the peptide cyclization, and the length and branching of the fatty acid chain [11]. Based on their structures, lipopeptides produced by *Bacillus* sp. are mainly classed into three categories: iturins, fengycins, and surfactins. Iturins are cyclopeptides composed of C_14_-C_17_ β-amino fatty acids and heptapeptides. Fengycins consist of β-hydroxy fatty acid chains and decapeptides, which form cyclic lactone rings. Surfactins contain cyclic lactone rings consisting of C_13_-C_16_ β-hydroxy fatty acids and heptapeptides [12]. Most *Bacillus* sp. can produce one type of lipopeptide and a few can produce two or three types of lipopeptides [13,14,15]. The biological activities of lipopeptides are closely related to the sequence of amino-acid residues, the cyclization of the peptide, and the length and branching of the fatty acid chain [16].

With the continuous extensive exploration of terrestrial microbial resources, marine microorganisms have gradually attracted scientists’ interests [17]. The marine environment has an extraordinary supply of natural bioactive products, many of which are demonstrated to have different structural or chemical characteristics from those found on land [18]. The deep-sea environment is a unique habitat, and deep-sea microorganisms, because of their adaptation to this extreme environment, have the potential to produce novel secondary metabolites with biological activities.

In this present study, we isolated a bacterial strain *Bacillus* sp. CS30 from the cold seep in deep sea, which can significantly inhibit the growth of the plant pathogen *Magnaporthe grisea*. We purified the active antifungal agents and determined their structural and functional properties. Furthermore, their antifungal mechanisms were also investigated.

## 2. Results

### 2.1. Isolation and Identification of Marine Bacterial Strain against M. grisea

In order to obtain potential bacterial strains producing antifungal agents, about 500 marine bacteria were isolated from the sediments of cold seep in deep sea, and their inhibitory activities against the plant pathogenic fungus *M. grisea* were determined. Among these, strain CS30 was shown to significantly inhibit the growth of *M. grisea*. The strain CS30 is a rod-shaped, Gram-positive, and spore-forming bacterium, and formed irregular and wrinkled colonies on LB medium. Genetic analysis by sequencing of the 16S rDNA (Accession no. MH793466) showed that strain CS30 shared a high degree of identity (99%) with *B. tequilensis* strain JS5L (Sequence ID: KX129852.1) and *B. subtilis* KACC15929 (Sequence ID: MK240359.1) in the NCBI database. Therefore, the marine bacterial strain CS30 was designated as *Bacillus* sp. CS30.

### 2.2. Purification of the Antifungal Agents Produced by Bacillus sp. CS30

**Figure 1:**
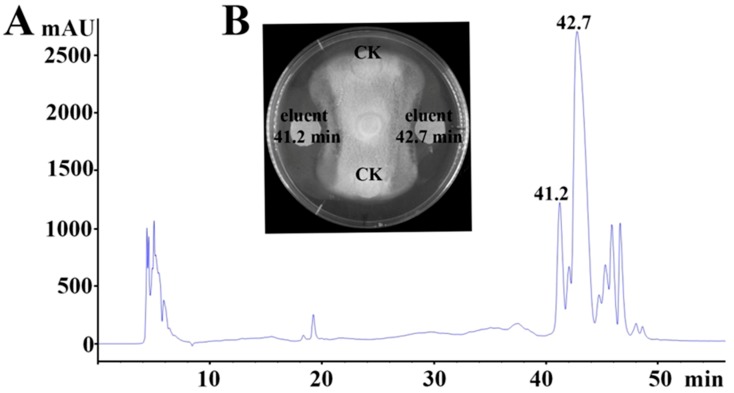
Purification and characterization of the antifungal agents produced by *Bacillus* sp. CS30 against *M. grisea*. (**A**) HPLC chromatogram of the antifungal agent produced by *Bacillus* sp. CS30. (**B**) Growth inhibition of *M. grisea* caused by the purified antifungal agents. Sterile filter paper disks containing the same amount of purified active agents and a methanol control are indicated. The active agents eluted at 41.2 min and 42.7 min in panel A are indicated as “eluent 41.2 min” and “eluent 42.7 min” in panel B, respectively.

The antifungal agents were purified by acidic precipitation, methanol extraction, sequential Sephadex LH-20, and RP-HPLC. As shown in Table 1, after acidic precipitation and methanol extraction, the specific activity against *M. grisea* was enhanced from 50 AU/mL to 320 AU/mL, and the yield recovery was 32%. The specific activity gradually increased as the purification steps continued. The specific activity reached 1200 AU/mL after separation on a Sephadex LH-20 column, and the corresponding yield was 19.2%. At the final purification step, two antifungal peaks were obtained, one antifungal fraction was eluted at 41.2 min (designated as eluent 41.2 min), and another antifungal fraction was eluted at 42.7 min (designated as eluent 42.7 min) (Figure 1), and the corresponding yield recoveries were 3% and 9%, respectively.

### 2.3. Mass Spectrometry Analysis of the Purified Antifungal Agents

**Figure 2:**
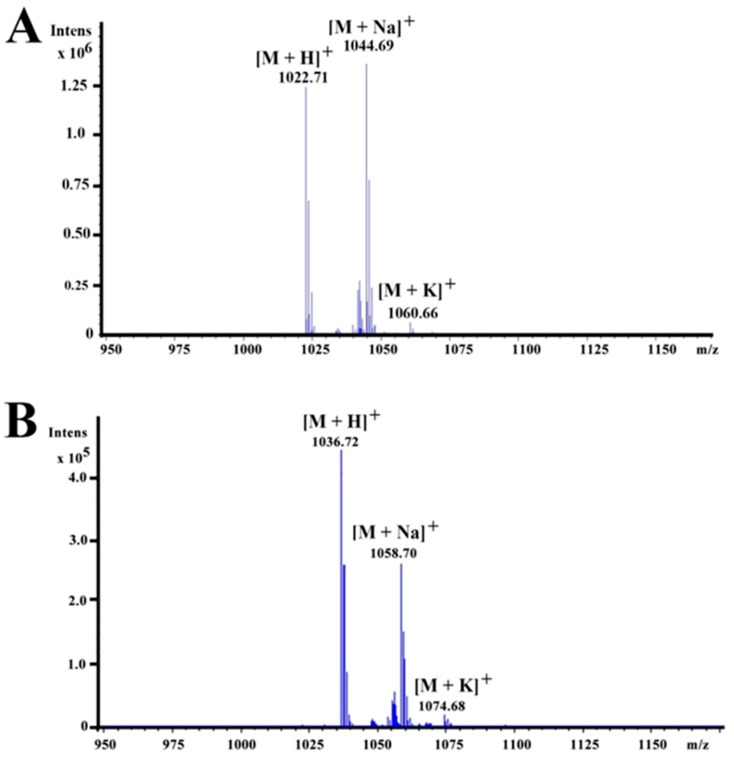
ESI-QTOF-MS analysis of the antifungal agents produced by *Bacillus* sp. CS30. (**A**) ESI-QTOF-MS analysis of antifungal agent eluted at 41.2 min in Figure 1. (**B**) ESI-QTOF-MS analysis of antifungal agent eluted at 42.7 min in Figure 1.

In order to determine the molecular mass of the purified bioactive agents, the purified fractions were analyzed by ESI-QTOF-MS. For the active fraction eluted at 41.2 min, three peaks appeared at *m*/*z* values of 1022.71, 1044.69, and 1060.66 (Figure 2A), which correspond to the single protonated agent [M + H]^+^, sodium-cationized ion [M + Na]^+^, and potassium-cationized ion [M + K]^+^, respectively. The results also showed the predicted molecular formula C_53_H_96_N_7_O_12._ For the active fraction eluted at 42.7 min, three peaks appeared at *m*/*z* values of 1036.72, 1058.70, and 1074.68 (Figure 2B), corresponding to the single protonated agent [M + H]^+^, sodium-cationized ion [M + Na]^+^, and potassium-cationized ion [M + K]^+^, respectively. The result also showed the predicted molecular formula C_54_H_98_N_7_O_12_. Based on the above results, we presumed that the antifungal fraction eluted at 42.7 min had one more methylene group (-CH_2_) than the antifungal fraction eluted at 41.2 min. Along with our purification scheme and previous reports about surfactin homologs, most of which were detected at *m*/*z* 1008, 1022, 1030, 1036, 1044, 1058, and 1072 [12,14,19], we deduced that the active agents produced by *Bacillus* sp. CS30 belonged to the category of surfactins.

### 2.4. Amino Acid Analysis of the Purified Antifungal Agent

**Figure 3:**
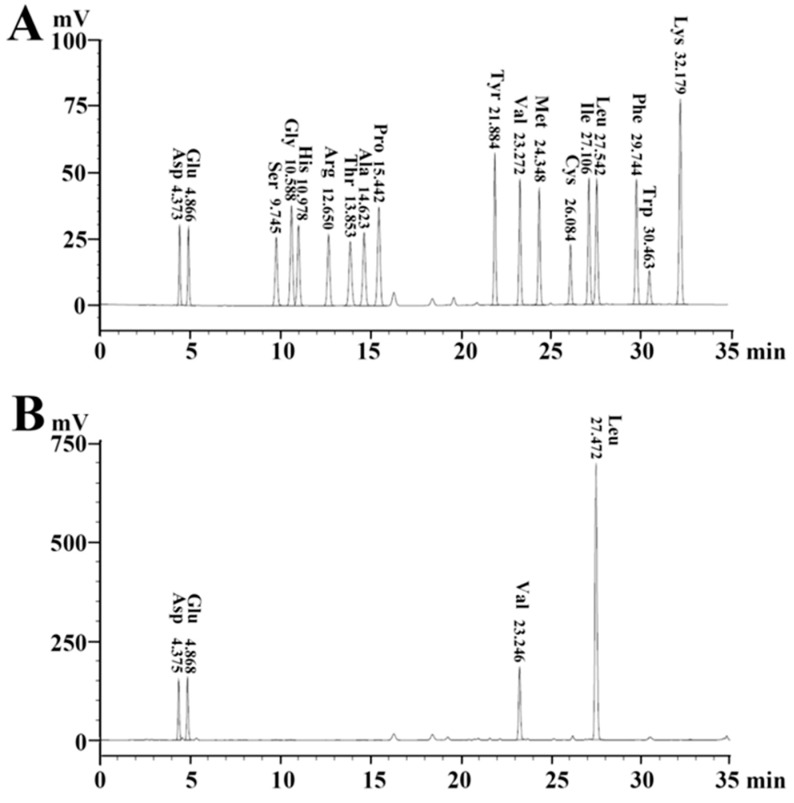
Chromatograms of phenylisothiocyanate derivatives of amino acids by C18 RP-HPLC. (**A**) Standard amino acids: Asp, Glu, Ser, Gly, His, Arg, Thr, Ala, Pro, Tyr, Val, Met, Cys, Ile, Leu, Phe, Trp, and Lys. (**B**) The hydrolyzed sample of antifungal agent eluted at 42.7 min in Figure 1.

Typical surfactins contain a heptapeptide, which is linked to a β-hydroxy fatty acid of 13–15 C atoms via a lactone bond, and different types of surfactins vary in the amino acids of the heptapeptides, the number of C atoms in the fatty acid chain, or structural conformation [1]. In order to resolve the nature of the amino acid components of the heptapeptide in our purified antifungal agents, the amino acids of the main active fraction eluted at 42.7 min were analyzed. As shown in Figure 3, only four kinds of amino acids, Glu, Asp, Val, and Leu, were detected, indicating that the heptapeptide of the antifungal agent contained Glu, Asp, Val, and Leu.

### 2.5. ESI-QTOF-Mass/Mass Analysis of the Purified Antifungal Agents

**Figure 4:**
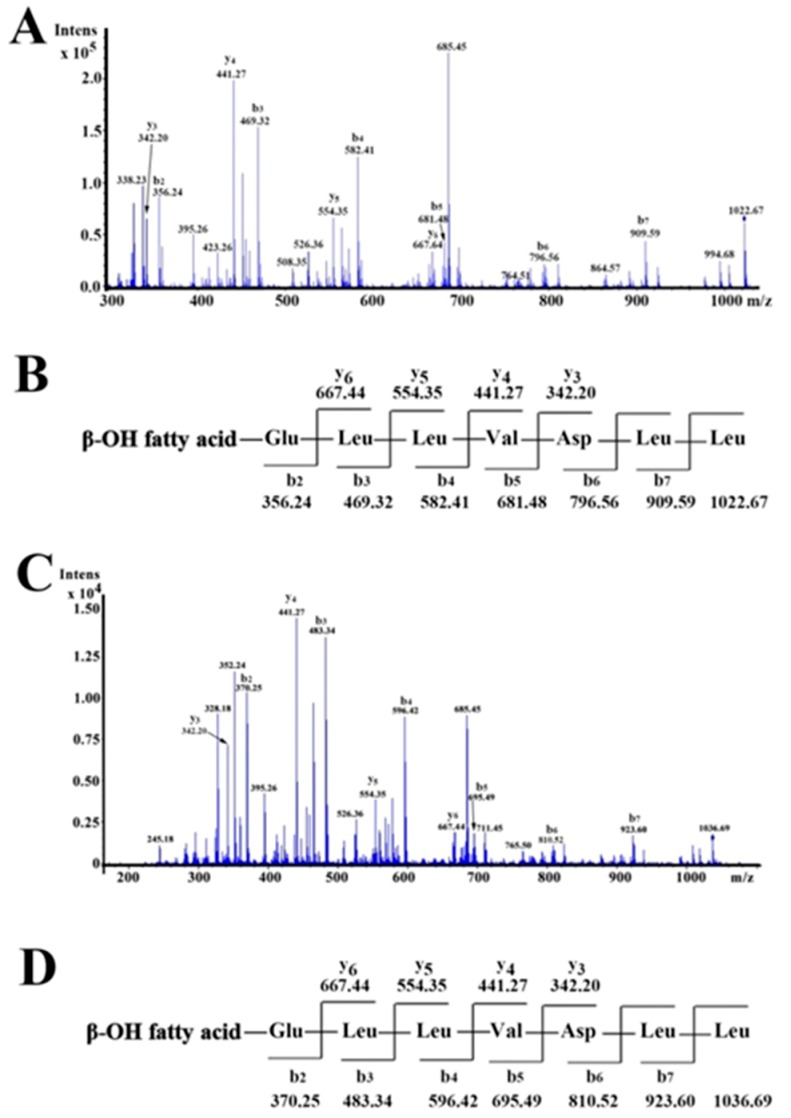
ESI-QTOF- MS/MS analysis of the antifungal agents eluted at 41.2 min (**A**,**B**) and 42.7 min (**C**,**D**), as shown in Figure 1, respectively.

To obtain the primary structure of the purified antifungal agents, the antifungal fractions eluted at 41.2 min and 42.7 min were also analyzed by ESI-QTOF-MS/MS, respectively. For the fraction eluted at 41.2 min, as shown in Figure 4A, the b and y fragments were generated by MS/MS analysis. Starting from the N-terminal, fragments of b ion in order were 909.59 (b_7_), 796.56 (b_6_), 681.48 (b_5_), 582.41 (b_4_), 469.32 (b_3_), and 356.24 (b_2_). Considering a value of 1022.67 for [M + H]^+^, the differences between the values were exactly the mass of ion fragments of Leu, Leu, Asp, Val, Leu, and Leu. Starting from the C-terminal, detectable fragments y ions in order were 667.44 (y_6_), 554.35 (y_5_), 441.27 (y_4_), and 342.20 (y_3_), and the differences between the values were the mass of ions fragments of Leu, Leu, and Val. Along with the results of the amino acids analysis, the primary amino acid sequence of the antifungal fraction eluted at 41.2 min was proposed to be β-OH fatty acid-Glu-Leu-Leu-Val-Asp-Leu-Leu from the N terminal to the C terminal (Figure 4B), so the antifungal fraction was then designated as surfactin CS30-1.

As for the fraction eluted at 42.7 min, the b and y fragments generated by MS/MS analysis are shown in Figure 4C. Starting from the N-terminal, fragments of b ion in order were 923.60 (b_7_), 810.52 (b_6_), 695.49 (b_5_), 596.42 (b_4_), 483.34 (b_3_), and 370.25 (b_2_). Considering 1036.69 for [M + H]^+^, the differences between the values were exactly the mass of ion fragments of Leu, Leu, Asp, Val, Leu, and Leu. Starting from the C-terminal, detectable fragments of y ions in order were 667.44 (y_6_), 554.35 (y_5_), 441.27 (y_4_), and 342.20 (y_3_), and the differences between the values were the mass of ion fragments of Leu, Leu, and Val. Therefore, the primary amino acid sequence of the antifungal fraction eluted at 42.7 min was proposed to be β-OH fatty acid-Glu-Leu-Leu-Val-Asp-Leu-Leu from the N terminus to the C terminus (Figure 4D), so the antifungal fraction was then designated as surfactin CS30-2.

### 2.6. Activity Assay of Surfactin CS30-1 and Surfactin CS30-2 against Plant Pathogen M. grisea

**Figure 5:**
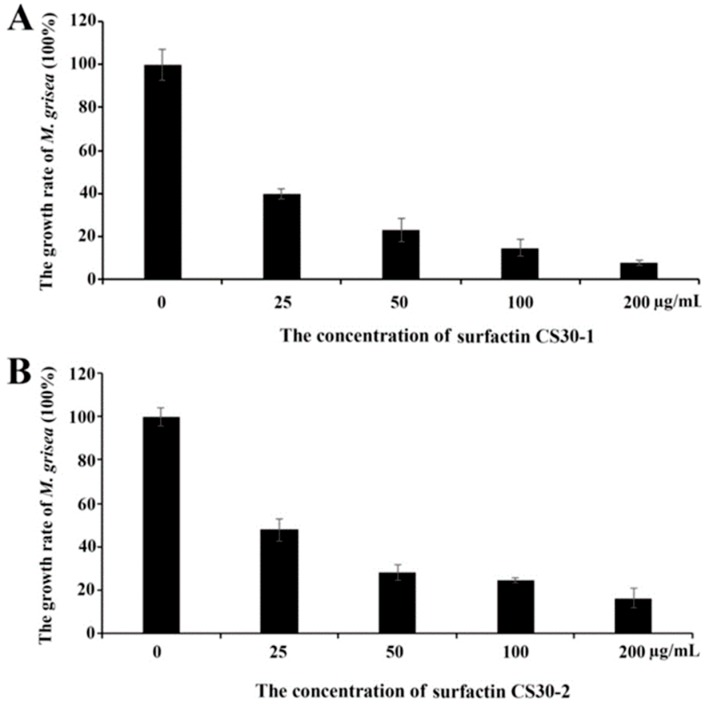
Growth inhibition of surfactin CS30-1 (**A**) and surfactin CS30-2 (**B**) against *M. grisea*. The experiments were performed in three replicates.

Though the antifungal activities of surfactins have been reported [20], the differences between different homologs have rarely been described. In order to investigate whether there is a difference in the activity of different surfactin homologs, the antifungal activities of the purified surfactin CS30-1 and surfactin CS30-2 were tested against *M. grisea*. As shown in Figure 5A, when treated with 25 µg/mL of surfactin CS30-1, the growth of *M. grisea* was decreased to 40%, and the growth of *M. grisea* was gradually reduced as the concentration of surfactin CS30-1 was increased. When the concentration of surfactin CS30-1 was increased to 200 µg/mL, the growth of *M. grisea* was decreased to about 10%. When treated with purified surfactin CS30-2, as shown in Figure 5B, the growth of *M. grisea* was also gradually reduced as the concentration of the surfactin CS30-2 was increased, but the growth of *M. grisea* was still higher than 20% when the concentration of surfactin CS30-2 was increased up to 200 µg/mL, indicating that the antifungal activity of surfactin CS30-1 was higher than that of surfactin CS30-2.

### 2.7. Ultrastructural and Morphological Changes of M. grisea Hyphae Caused by Surfactin CS30-1 and Surfactin CS30-2

**Figure 6:**
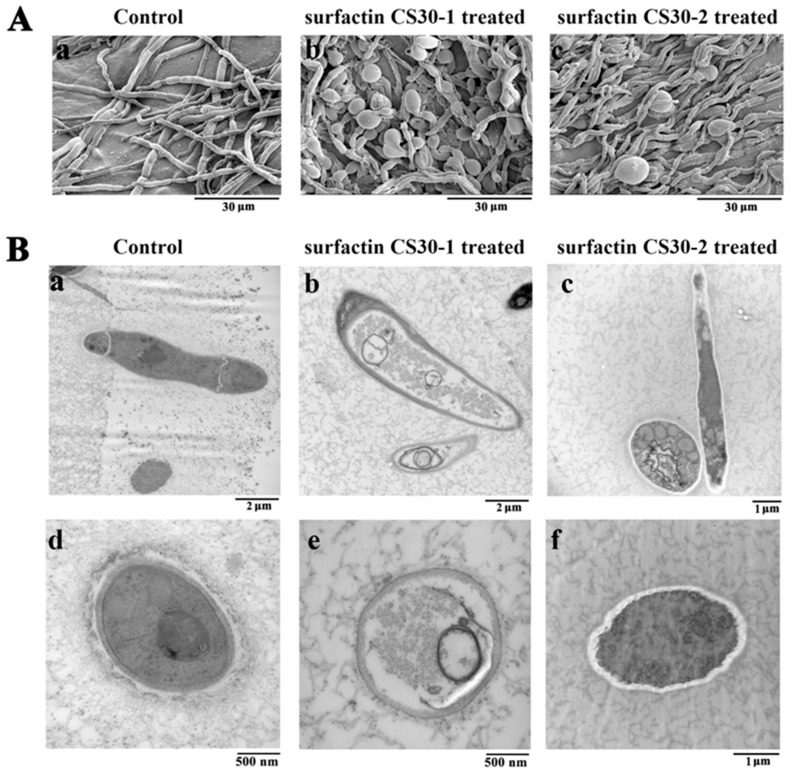
Effects of surfactin CS30-1 and surfactin CS30-2 on the morphology and ultrastructure of *M. grisea* hyphae observed by SEM (**A**) and TEM (**B**). In the control groups, *M. grisea* hyphal cells were treated with the same amount of methanol, while in the tested groups, *M. grisea* hyphal cells were treated with 20 μL of 200 μg/mL surfactin CS30-1 or surfactin CS30-2 dissolved in methanol.

In order to investigate the antifungal mechanism of surfactin CS30-1 and surfactin CS30-2 against fungal hyphae, *M. grisea* was treated with the same concentration of surfactin CS30-1 and surfactin CS30-2, respectively, and was then observed under SEM and TEM. As shown in Figure 6A, the hyphae of *M. grisea* in the control group grew normally with a straight appearance under SEM, while the hyphae appeared swollen and irregular after exposure to surfactin CS30-1 and surfactin CS30-2. Furthermore, when treated with surfactin CS30-1, the ratio of the swollen hyphae is higher than that of swollen hyphae treated with surfactin CS30-2. When observed under TEM, as shown in Figure 6B, the hyphae in the control group showed a smooth surface, distinct cell wall, intact plasma membranes and septum, and a uniformly distributed cytoplasm (Figure 6B, panels a and d). On the contrary, when treated with surfactin CS30-1, the cell wall of the hyphae became thinner than that in the control group, and the cell membrane was incomplete. In addition, the cytoplasm appeared sparse compared with that in the control group (Figure 6B, panels b and e), indicating a serious leaking of the cytoplasm. When treated with surfactin CS30-2, the cell wall of the hyphae became swollen and appeared bright, and the cytoplasm was unevenly distributed (Figure 6B, panels c and f). It is obvious that the cytoplasm in hyphae treated with surfactin CS30-1 was more sparse than that treated with surfactin CS30-2, indicating more serious cytoplasm leaking when treated with surfactin CS30-1. Based on the results of SEM and TEM, we concluded that surfactin CS30-1 exhibited higher antifungal activity than surfactin CS30-2, which is coincident with the results of the activity assay.

### 2.8. Accumulation of ROS and Cell Death of M. grisea Caused by Surfactin CS30-1 and Surfactin CS30-2

**Figure 7:**
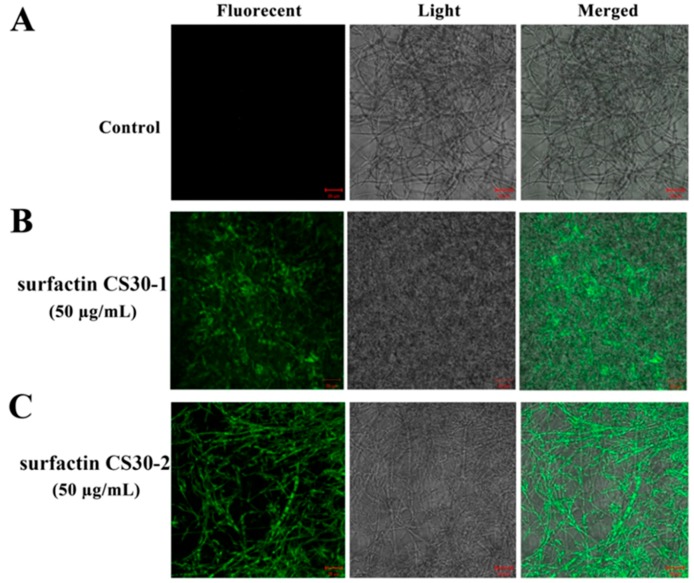
ROS detection in *M. grisea* hyphal cells observed under light microscopy and fluorescence microscopy in the control group (**A**) and the tested group treated with surfactin CS30-1 (**B**) and surfactin CS30-2 (**C**), respectively. In the control group, *M. grisea* hyphal cells were treated with the same amount of methanol, while in the tested group, *M. grisea* hyphal cells were treated with 50 μg/mL surfactin CS30-1 (**B**) or surfactin CS30-2 (**C**) dissolved in methanol. Scale bar = 20 µm.

A dramatic increase of ROS could cause irreversible oxidative damage to the cell, thus leading to cell death of the fungus [21,22]. Since surfactin CS30-1 and surfactin CS30-2 can cause serious damage to the fungus, in order to investigate whether ROS take part in this process, intracellular ROS was detected by the fluorescent probe 2′,7′-dichlorofluorescin diacetate (DCFH2-DA). As shown in Figure 7, when treated with 50 µg/mL surfactin CS30-1 and surfactin CS30-2, respectively, *M. grisea* hyphae exhibited strong green fluorescence (Figure 7B,C), while green fluorescence was hardly detected in the hyphae of the control group (Figure 7A), indicating that the generation of ROS in hyphae of *M. grisea* was dramatically increased after treatment with surfactin CS30-1 and surfactin CS30-2, respectively.

**Figure 8:**
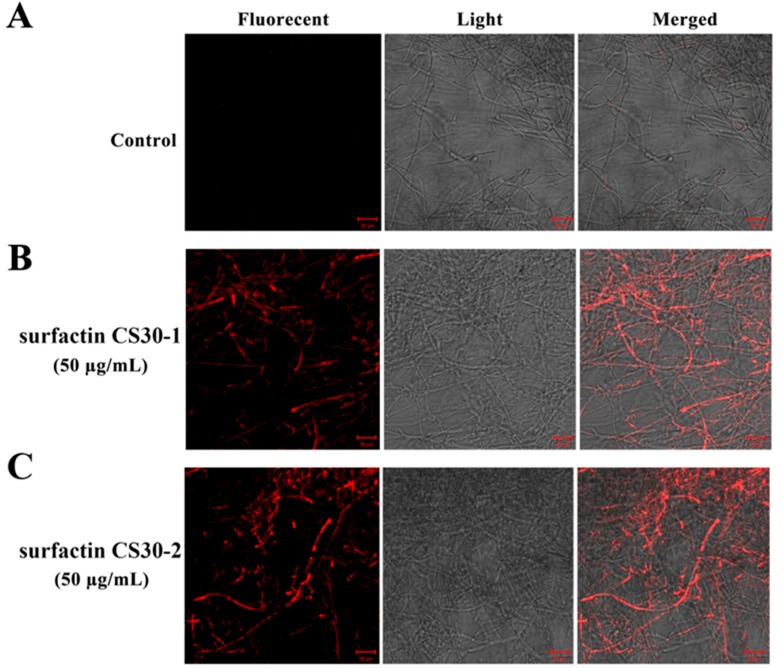
Effect of surfactin CS30-1 and surfactin CS30-2 on the viability of *M. grisea* observed under light microscopy and fluorescence microscopy in the control group (**A**) and the tested groups treated with surfactin CS30-1 (**B**) and surfactin CS30-2 (**C**), respectively. In the control groups, *M. grisea* hyphal cells were treated with the same amount of methanol, while in the tested group, *M. grisea* hyphal cells were treated with 50 μg/mL surfactin CS30-1 (B) or surfactin CS30-2 (C) dissolved in methanol. Scale bar = 20 µm.

To further investigate whether the increased generation of ROS caused damage to *M. grisea*, the cells analyzed by the fluorescent probe DCFH2-DA were washed gently, and then stained with propidium iodide to analyze whether cell death had occurred. Propidium iodide fluoresces red in response to membrane damage and is used as an indicator of the presence of dead cells. As shown in Figure 8, strong red fluorescence was observed in the hyphae treated with surfactinCS30-1 and surfactin CS30-2 (Figure 8B,C), while the hyphae in the control group exhibited no visible red fluorescence (Figure 8A), indicating that the increased generation of ROS in *M. grisea* was accompanied by cell death after treatment with surfactin CS30-1 or surfactin CS30-2.

### 2.9. Effect of Surfactin CS30-1 and Surfactin CS30-2 on the Plant Infection of M. grisea

**Figure 9:**
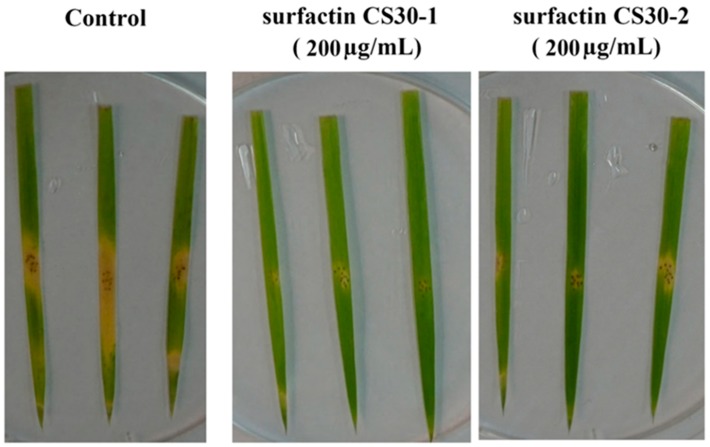
Effects of surfactin CS30-1 and surfactin CS30-2 on the plant infection of *M. grisea*. In the control groups, *M. grisea* hyphal cells were treated with the same amount of methanol, while in the tested group, *M. grisea* hyphal cells were treated with 200 μg/mL surfactin CS30-1 or surfactin CS30-2 dissolved in methanol.

Since surfactin CS30-1 and surfactin CS30-2 can obviously inhibit the growth of *M. grisea*, which can infect rice, resulting in serious disease, the leaves of rice seedlings were used to evaluate whether surfactin CS30-1 and surfactin CS30-2 can reduce the pathogenicity of *M. grisea* on the plant. As shown in Figure 9, after inoculation of *M. grisea*, the lesion starts from the wounded spot and expands to the surrounding area in the control group, while when treated with surfactin CS30-1 and surfactin CS30-2, the lesions are much smaller than those in the control group, and the expansion to the surrounding area is reduced, indicating that both surfactin CS30-1 and surfactin CS30-2 have promising potential to biocontrol plant disease.

## 3. Discussion

Rice (*Oryza sativa*) is one of the most important food crops in the world, and rice blast disease caused by the fungus *M. grisea*, is one of the most devastating cereal diseases due to the critical decrease of rice yield and quality [23]. One common solution to control the pathogen is the use of synthetic chemical agents; however, excessive use of chemical agents often results in environmental problems by inducing the emergence of resistant pathogens and leaving fungicide deposits on food. Lipopeptides produced by the *Bacillus* genus are mostly exploited agents to biocontrol plant diseases because of their low toxicity and high degradability [11,24]. Their protective effect may rely on directly antagonizing pathogen growth, or indirectly protecting plants as elicitors to induce systemic resistance in plants [25]. To obtain potential agents against *M. grisea*, the screening of marine bacteria was performed via targeting this fungus. *Bacillus* sp. CS30 derived from deep sea was found to significantly inhibit the growth of *M. grisea*, and its metabolite surfactin CS30-1 and surfactin CS30-2 can obviously reduce the pathogenicity of *M. grisea* on plants. Therefore, surfactin CS30-1 and surfactin CS30-2 are ideal candidates of fungicide to biocontrol blast disease of rice caused by *M. grisea*.

Surfactins are important categories of lipopeptides produced by *Bacillus* sp., which are not only very popular in the food industry as emulsifier, foaming, and antiadhesive agents, but are also widely reported in the agriculture and pharmaceutics field as antimicrobial, anticancer, and antiviral agents [26]. Surfactins are well-known for their antibacterial activities against pathogenic bacteria, such as *Pseudomonas aeruginosa*, *Streptococcus aureus*, *Salmonella typhi*, and *Shigella flexneri* [17]. Compared with antibacterial activity, only a few types of surfactins have been reported to exhibit marked toxicity against fungi [26,27,28,29]. Though surfactin A and its homologs have recently been reported to possess antifungal activity [20], differences in the antifungal activity and mechanisms of the various homologs have not been described. In our study, the antifungal activity of surfactin CS30-1 is higher than that of surfactin CS30-2, and the morphology changes of *M. grisea* hyphae caused by surfactin CS30-1 and surfactin CS30-2 are different. Furthermore, we also observed that surfactin CS30-1 had no obvious antitumor activity against human liver cancer cell Huh7.5, while surfactin CS30-2 had strong antitumor activity, indicating that although the amino acid sequences of surfactin CS30-1 and surfactin CS30-2 are identical, their antifungal activity and antitumor activity are different because of the different β-hydroxy fatty acid chains.

High concentrations of ROS are harmful to cells and result in cell death [30,31]. It is reported that lipopeptides exert biological functions against plant pathogens by inducing ROS production [21,22]. In our study, increased generation of ROS was also observed when the plant pathogen *M. grisea* was treated with surfactin CS30-1 and surfactin CS30-2. In addition, the increased generation of ROS was accompanied by cell death when treated with surfactin CS30-1 and surfactin CS30-2, indicating that the increased generation of ROS induced by surfactin CS30-1 and surfactin CS30-2 is an important factor that causes cell death. Furthermore, we noticed that the cell wall became thinner and the cytoplasm became sparse when treated with surfactin CS30-1, while the cell wall became swollen and bright and cytoplasm was distributed unevenly when treated with surfactin CS30-2, indicating that although the ultrastructural and morphological changes caused by surfactin CS30-1 and surfactin CS30-2 are different, both of them caused serious damage to the cell wall and cytoplasm. However, the exact mechanisms of surfactin CS30-1 and surfactin CS30-2 causing fungal cell death still need to be further investigated.

Altogether, two surfactin homologs surfactin CS30-1 and surfactin CS30-2 were isolated and purified from the marine bacterium *Bacillus* sp. CS30. Though the antifungal activity and antifungal mechanism are different, both of them have strong antifungal activity, indicating that they have the potential to biocontrol plant disease caused by *M. grisea* in the future.

## 4. Materials and Methods

### 4.1. Strain Isolation, Culture Conditions, and Strain Identification

Sediments were collected from the Formosa ridge during the cold seep cruise of the R/V *Kexue* in South China sea on October of 2017 (119°17′09.655″ E, 22°06′55.169″ N). The sediments were diluted with a series of 2216E broth medium (5 g/L peptone, 1 g/L yeast extract, 1 L filtered seawater, pH adjusted to 7.4–7.6), and then spread plated on 2216E agar medium and cultured at 28 °C. The single colonies were further screened for their ability to produce antifungal agents. The fungal strain *M. grisea* used in this study was incubated onto potato dextrose agar (PDA) and incubated at 28 °C. For the strain identification, genomic DNA was extracted from the isolate, and universal primers 27F (AGAGTTTGATCCTGGCTCAG) and 1541R (AAGGAGGTGATCCACCC) were used to amplify the 16S rDNA gene sequence. The 16S rDNA sequence was analyzed by using the BLAST programs in the NCBI database (https://blast.ncbi.nlm.nih.gov/Blast.cgi) to identify the strain.

### 4.2. Screening of Bacteria Producing Antifungal Agent

To screen bacteria inhibiting the growth of fungi, the procedure was carried out as Zhang et al. described, with little modification [21]. Briefly, the mycelium plugs (diameter, 10 mm) were taken out from the freshly cultured *M. grisea* and placed at the centers of new PDA plates. A total of 10 µL of different bacteria was seeded 3 cm away from the mycelium plug margin of *M. grisea* and incubated at 28 °C. The fungal growth zone was measured to detect whether the bacteria produced antifungal agents after incubation for three days.

### 4.3. Isolation and Purification of the Antifungal Agent

To obtain the active agent inhibiting the growth of fungal cells, the procedure was carried out according to the method described by Xiu et al., with little modification [32]. Briefly, the strain *Bacillus* sp. CS30 was cultured in a 250-mL glass flask filled with Luria Bertani (LB) medium (10 g/L peptone, 5 g/L yeast extract, 10 g/L NaCl, pH adjusted to 7.0) at 28 °C with shaking at a speed of 160 rpm. The cell culture was centrifuged at 8000× *g* for 10 min after being incubated for two days, and the supernatant was then adjusted to a pH of 2.5 using a 6 N HCl solution and kept at 4 °C overnight. To obtain the precipitate, the supernatant was collected by centrifugation at 8000× *g* at 4 °C for 10 min and washed with 0.1 N HCl. The active agent was extracted from the precipitate by gradually increasing the concentration of methanol, and was then loaded onto a Sephadex LH-20 column (GE, Hartford, CT, USA) and eluted with methanol as the mobile phase at the flow rate of 1.0 mL/min. Each eluted fraction was spotted on a small sterile paper disc to detect its antifungal activity against *M. grisea*. Further purification of the bioactive agent was achieved via RP-HPLC (Agilent 1260 Infinity, Santa Clara, CA, USA) on an Eclipse XDB-C18 column (5 µM, 250 × 9.4 mm, Agilent). The column was eluted with a linear gradient of 80% to 100% methanol over 45 min at a flow rate of 2.0 mL/min, and then eluted with 100% methanol until 60 min. Elution was monitored using a UV detector set at 230 nm, and fractions of each eluted peak were tested for their biological activity against *M. grisea*.

### 4.4. Activity Assay of the Purified Antifungal Agent against M. grisea

To detect the antifungal activity of the purified antifungal agent on PDA medium, 20 µL of the purified samples was spotted on a small sterilized filter paper and placed 2 cm away from the margin of freshly grown *M. grisea*. One arbitrary unit (AU) was defined as the reciprocal of the highest dilution yielding a definite inhibition against *M. grisea* on PDA medium. To detect the inhibition rate of the purified antifungal agent against *M. grisea*, the experiment was performed as described by Romano et al., with minor modification [27]. Briefly, the bioassay was conducted in a 96-well plate, which contained different concentrations of purified bioactive agent and freshly cultured *M. grisea* conidia. Plates were incubated at 28 °C on a rotary shaker (160 rpm) for two days, and the fungal growth was determined spectrophotometrically at 595 nm by a microplate reader (Infinite M1000 Pro, TECAN, Mannedorf, Switzerland). Relative fungal growth of *M. grisea* treated with the purified bioactive agent was normalized to the growth of *M. grisea* in the control group treated with equal amounts of methanol. All experiments were performed in triplicate.

### 4.5. Amino Acid Analysis of the Purified Antifungal Agent

To obtain the amino acid component of the purified antifungal agent, the active fraction of the main peak was harvested from its HPLC fraction and dried by vacuum freezing. The dried sample was hydrolyzed with 1 mL of 6 M HCl at 110 °C for 24 h and then dried under vacuum to remove residual HCl, and the dried sample was dissolved in 1 mL double-distilled water. The analysis of amino acids was carried out as the method described previously, with minor modification [12,33]. Briefly, eighteen amino acids (Sigma-Aldrich, Saint Louis, MI, USA) were used for standard reference: Asp, Glu, Ser, Gly, His, Arg, Thr, Ala, Pro, Tyr, Val, Met, Cys, Ile, Leu, Phe, Trp, and Lys. A 200 μL aliquot of the 18 standard amino acids (2.5 mM) or the re-dissolved hydrolysis sample solution was mixed with 100 μL of 0.1 M phenylisothiocyanate and 1 M triethylamine in acetonitrile. Then, the mixture was vortexed for 1 min and incubated for 60 min at room temperature. After derivatization, 400 μL of n-hexane was added and the mixture was vortexed for 1 min and allowed to stand for 5 min. The bottom acetonitrile phase was collected and further analyzed by HPLC on C18 column (5 µM, 250 × 4.6 mm, Agilent). The mobile phases were as follows: 0.1 M sodium acetate (pH 6.5, adjusted with acetic acid)–acetonitrile (A; 93:7, v/v) and acetonitrile–water (B; 4:1, v/v). The proportion of the mobile phases was controlled using the following gradient program: 0 min, 0 % mobile phase B; 11 min, 7 % mobile phase B; 13.9 min, 12 % mobile phase B; 14 min, 15 % mobile phase B; 29 min, 34% mobile phase B; 32 min, 70 % mobile phase B; 35 min, 100 % mobile phase B; 42 min, 100 % mobile phase B; 45 min, 0 % mobile phase B; and 60 min, 0 % mobile phase B. The mobile phase was kept at a flow rate of 1.0 mL/min, and the chromatograms were detected at 254 nm.

### 4.6. Structural Elucidation of the Antifungal Agent

To obtain the molecular weight of the purified antifungal agent, electrospray ionization and quadrupole time-of-flight mass spectrometry (ESI-QTOF-MS) analysis with a Bruker maXis mass spectrometer (Bruker, Berlin, Germany) was conducted. Data from ESI-QTOF-MS were acquired in positive ion mode under the following conditions: 3200-V capillary voltage, 4.0 L/min dry gas, and 200 °C dry gas temperature. To elucidate the primary peptide sequence of the purified bioactive agent, ESI-QTOF-MS/MS analysis was also performed on this instrument using the multiple reaction monitoring (MRM) mode as previously described [34,35].

### 4.7. Ultrastructural and Morphological Observation of Fungal Hyphae Caused by the Purified Antifungal Agent

To observe the morphological changes of *M. grisea* hyphae caused by the purified antifungal agent, 20 µL of 200 µg/mL purified bioactive agent was spotted on a small sterilized filter paper and placed 2 cm away from the margin of freshly grown *M. grisea*. The mycelium margin of *M. grisea* was cut out and prefixed with 2.5% glutaraldehyde for 1 h, after being further incubated for two days, and was then rinsed three times for 10 min with 10 mM phosphate buffer and dehydrated through an ethanol gradient. As for the control group, 20 µL methanol was spotted on a small sterilized filter paper and the following procedure was carried out as described for purified bioactive agents. For scanning electron microscopy (SEM) observation, the samples were observed at 5 kV with SEM (S-3400N; Hitachi, Tokyo, Japan). For the transmission electron microscopy (TEM) observations, samples were embedded in Epon 812, and ultrathin sections were collected and observed at 120 kV with TEM (HT7700; Hitachi).

### 4.8. ROS Detection in Fungal Cells Induced by the Purified Antifungal Agent

For ROS detection in plant pathogen *M. grisea* induced by the purified antifungal agent, freshly diluted conidial suspensions of *M. grisea* were treated with 50 µg/mL purified bioactive agent and incubated at 28 °C for two days, washed in 10 mM phosphate buffer, and stained with 10 µM DCFH2-DA for 20 min at 37 °C in the dark [36,37]. The hyphal cells were observed and recorded under a fluorescence microscope with a filter (488 nm/525 nm) in the dark. In order to detect whether the cell death of *M. grisea* occurred after being treated with a purified antifungal agent, the cells analyzed by fluorescent probe DCFH2-DA were washed gently and stained with propidium iodide and observed under a fluorescence microscope with a filter of (535 nm/615 nm) [38]. For the control group, the cells were treated with equal amounts of methanol, and the following procedure was carried out as described for a purified antifungal agent.

### 4.9. Effects of Purified Antifungal Agent on the Plant Infection of M. grisea

In order to detect whether purified antifungal agent could reduce the pathogenicity of *M. grisea* on plants, the leaves of rice seedlings were cut off and sterilized with 75% ethanol, and then placed on filter paper, which was placed in a petri dish and soaked with adequate sterile distilled water. To facilitate the infection of *M. grisea*, the leaves were punctured gently with a syringe. Before inoculation, the wounded spot was sprayed with 200 µg/mL of purified bioactive agent. For the control group, the wounded spot was sprayed with the same amount of methanol. After that, the freshly prepared *M. grisea* was inoculated onto the wounded spot. The petri dishes containing the leaves of rice seedlings were incubated at room temperature and the pathogen lesions were observed after inoculation three days later. Each treatment used three leaves of rice seedlings.

## Figures and Tables

**Table 1:** Recoveries of two antifungal agents at different purification steps.

Purification Step	Volume (mL)	Specific Activity (AU/mL)	Total Activity (AU)	Recovery (%)
Growth medium	1000	50	5 × 10^4^	100%
Methanol extraction	50	320	1.6 × 10^4^	32%
Sephadex LH-20	8	1200	9.6 × 10^3^	19.2%
RP-HPLC eluent 41.2 min	1	1500	1.5 × 10^3^	3%
RP-HPLC eluent 42.7 min	1.5	3000	4.5 × 10^3^	9%
